# Harnessing Cation–Anion Synergistic Effect for High‐Performance Aqueous Zinc‐Ion Batteries

**DOI:** 10.1002/anie.3172435

**Published:** 2026-07-02

**Authors:** Weichen Li, Jiyang Liu, Junhong Guo, Anyao Song, Suli Chen, Ruwei Chen, Yunpeng Zhong, Yongkang Xing, Jihao Sun, Zhihong Tian, Guanjie He

**Affiliations:** ^1^ Engineering Research Center for Nanomaterials Henan University Kaifeng P. R. China; ^2^ Department of Chemistry University College London London UK; ^3^ Key Laboratory of Synthetic and Biological Colloids, Ministry of Education, School of Chemical and Material Engineering Jiangnan University Wuxi P. R. China

**Keywords:** aqueous zinc‐ion batteries, cation–anion synergistic effect, electrolyte additive, solid electrolyte interphase, zinc anode

## Abstract

Aqueous zinc‐ion batteries (AZIBs) are promising for large‐scale energy storage due to environmental friendliness, inherent safety, and low cost. However, the practical deployment of AZIBs is hindered by notorious side reactions at the zinc (Zn) anode, which seriously deteriorate the battery stability and reversibility. Here, we propose guanidine sulfate (GS) as an electrolyte additive, leveraging a cation–anion synergistic mechanism to jointly inhibit side reactions. Both theoretical and experimental results confirm that the guanidine cation (CH_6_N_3_^+^) preferentially adsorbs on the Zn anode surface, providing an electrostatic shielding effect that promotes uniform Zn deposition. Concurrently, the sulfate anion (SO_4_^2–^) contributes to the formation of a robust solid electrolyte interface (SEI), effectively inhibiting dendrite growth and enhancing interfacial stability. Consequently, Zn||Cu asymmetric cells with GS deliver a high Coulombic efficiency of 99.6% over 1200 cycles, while Zn||Zn symmetric cells exhibit an extended lifespan exceeding 500 h at various current densities. Furthermore, the Zn||O_d_‐NVO·nH_2_O full cells demonstrate outstanding cycling stability, retaining over 90% of initial capacity after 2000 cycles at current densities of 2 and 5 A g^−1^. This research provides a viable electrolyte design strategy leveraging cation–anion synergy, offering new insights into electrolyte modulation and advancing the performance of AZIBs.

## Graphical abstract

Guanidine (CH_6_N_3_^+^) cations preferentially adsorb on the Zinc (Zn) anode surface, generating an electrostatic shielding effect to promote uniform Zn^2+^ deposition. Meanwhile, SO_4_^2–^ anions participate in constructing the solid electrolyte interface (SEI) on the Zn anode, forming a robust protective layer. The cation–anion synergistic effect significantly enhances the performance of aqueous zinc‐ion batteries.

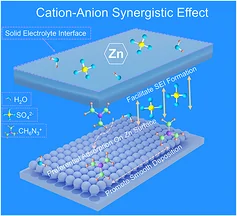

## 1 Introduction

The pursuit of green and sustainable energy storage technologies is essential for the continued advancement of industrial societies. Although lithium‐ion batteries (LIBs) have achieved a significant level of maturity in applications such as electric vehicles, consumer electronics, and various energy storage systems, their commercial prospects are increasingly constrained by limited lithium resources, high costs, and inherent safety risks [1, 2]. Compared with LIBs, aqueous zinc‐ion batteries (AZIBs) offer several benefits, including the natural abundance, low redox potential (−0.762 V vs. standard hydrogen electrode, SHE), and high theoretical capacity (820 mAh g^−1^ or 5855 mAh cm^−3^) of zinc (Zn) anode, along with the intrinsic safety of aqueous electrolytes [3, 4, 5, 6]. These benefits collectively contribute to the cost‐effectiveness and enhanced safety profile of AZIBs, positioning them as promising candidates for next‐generation energy storage. Nevertheless, the widespread implementation of this technology is still restricted by the poor reversibility and stability induced by severe side reactions, including the hydrogen evolution reaction (HER), chemical corrosion, and dendrite growth on the Zn anode [7, 8, 9].

To address the issues of poor reversibility and stability in AZIBs, various strategies have been proposed. Among these, facile electrolyte regulation offers distinct advantages over labor‐intensive cathode and anode modifications. Extensive studies have focused on regulating the solvation structure through cosolvents to reduce the number of active water molecules surrounding Zn^2+^, thus enhancing the electrochemical efficiency of AZIBs [10, 11]. Additionally, incorporating electrolyte additives into the electrolyte can form a shielding effect or regulate the electric double layer (EDL) structure, improving the stability and reversibility of the Zn anode [12, 13, 14, 15]. Furthermore, numerous researchers have been dedicated to creating an artificial solid electrolyte interface (SEI) to avoid direct interaction between the Zn anode and the bulk electrolyte, thereby alleviating side reactions. Nevertheless, ex‐situ SEI layers are prone to crack during cycling, leading to performance degradation or even battery failure. In contrast, in situ SEI layers, generated through reactions between electrolyte additives and the Zn anode during operation, offer a more robust and cost‐effective approach to stabilizing the Zn/electrolyte interface [16, 17, 18, 19, 20, 21].

Among various electrolyte additives, ionic electrolyte additives have attracted significant attention on account of their simple structures and low cost. For example, Zhang's group proposed Bmim^+^ as a functional additive and clearly elucidated its cation adsorption mechanism [22]. Similarly, Song's group explored the effects of SDB^−^ additive in improving AZIBs performance, demonstrating that the anion contributes to the suppression of side reactions [23]. Both cationic and anionic additives have been shown to regulate the solvation structure, enhance electrostatic shielding effects, and promote the formation of the SEI layer in AZIBs [24, 25, 26, 27]. However, despite their promising benefits, most studies tend to focus solely on the effects of individual anions or cations, while only a few have explored the synergistic effects of cations and anions [28]. A systematic understanding of their combined roles remains lacking. In light of this, it is necessary to identify and develop novel electrolyte additives that exhibit cation‐anion synergistic functionality, which could open new avenues for optimizing electrolyte design and enhancing AZIBs' performance.

Herein, guanidine sulfate (GS) is introduced as a novel ionic additive. Unlike recently reported guanidine‐based electrolyte systems that primarily focus on regulating bulk electrolyte chemistry and solvation structures [29, 30], this work presents a new electrolyte design paradigm based on the intrinsic cation–anion synergy within a single additive. Specifically, the guanidinium cation (CH_6_N_3_^+^), featuring a unique planar conjugated structure with delocalized positive charge and multiple nitrogen‐containing adsorption sites, preferentially adsorbs onto the Zn anode surface, forming an electrostatic shielding effect that effectively guides uniform Zn^2+^ deposition and inhibits dendrite formation. Meanwhile, the sulfate radical (SO_4_^2–^) participates in the formation of zinc hydroxide sulfate (ZHS), which contributes to a solid protective layer on the Zn anode, greatly preventing water‐induced side reactions. Through the synergistic efforts of CH_6_N_3_^+^ and SO_4_^2–^, the stability and reversibility of AZIBs are significantly enhanced. As a result, the assembled Zn||Cu cells demonstrate a high average Coulombic efficiency (CE) of 99.6% over 1200 cycles, significantly higher than that of the bare electrolyte. The assembled Zn||Zn symmetric cells operate stably for over 500 h under varying current densities, highlighting excellent long‐term cycling stability. Additionally, the Zn||O_d_‐NVO·nH_2_O full cells exhibit outstanding rate performance and maintain over 90% of their initial capacity after 2000 cycles at current densities of 2 and 5 A g^−1^, respectively. These findings underscore the effectiveness of cation–anion synergistic regulation as a practical strategy for enhancing the electrochemical performance and durability of AZIBs.

## 2 Results and Discussion

**FIGURE 1:**
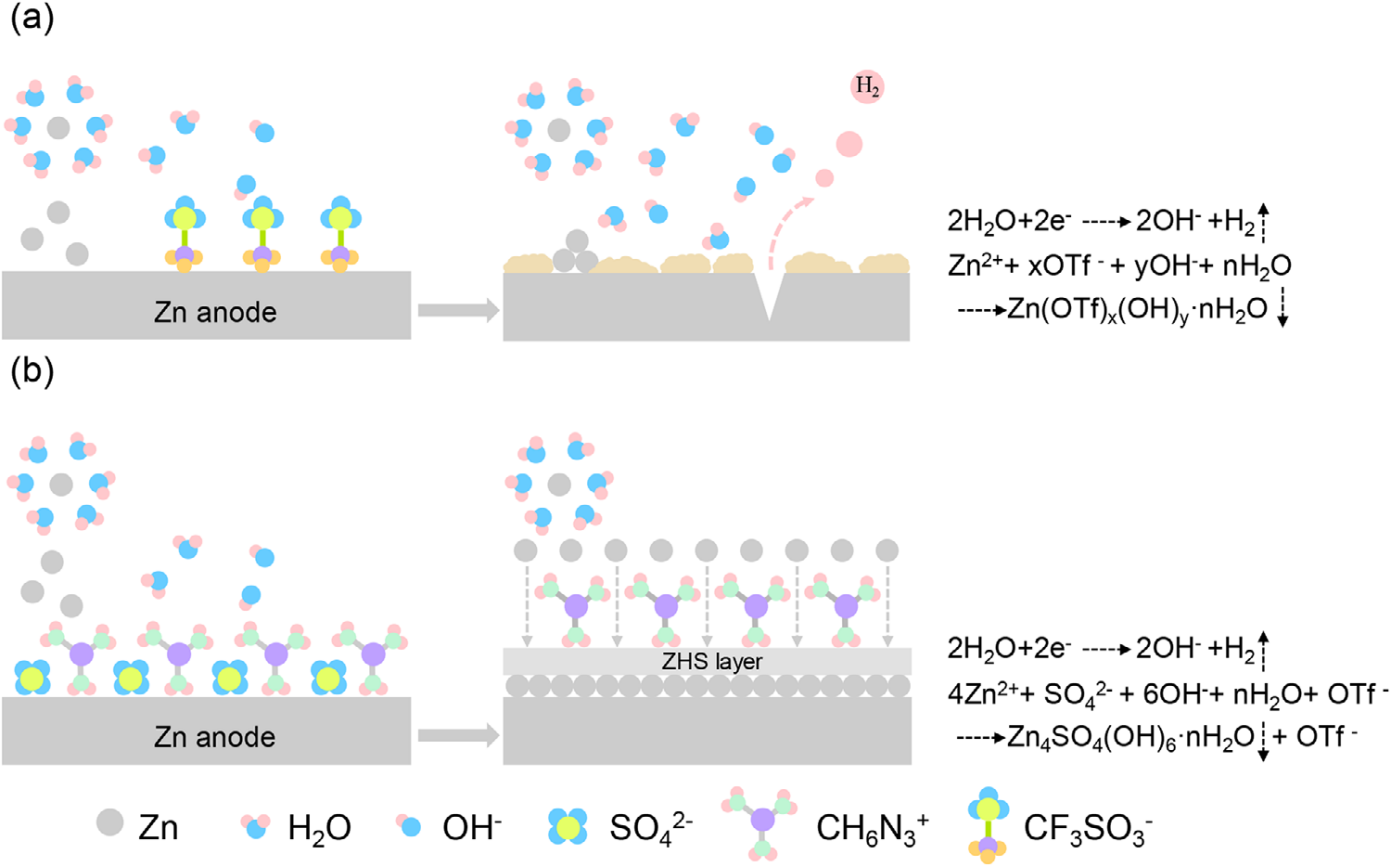
Schematic illustration of Zn plating behavior in (a) Zn (CF_3_SO_3_)_2_ electrolyte, (b) Zn (CF_3_SO_3_)_2_ electrolyte with GS additive.

Zn metal is thermodynamically unstable in a weakly acidic Zn (CF_3_SO_3_)_2_ electrolyte (ZT). The HER accompanied by the increased local pH value leads to self‐corrosion and the formation of Zn(OTf)_x_(OH)_y_·nH_2_O (ZTH) by‐products, which in turn hinder ion transport and contribute to uncontrolled dendrite growth, severely affecting the overall performance of AZIBs (Figure 1a) [31, 32, 33]. As illustrated in Figure 1b, the introduction of a functional GS additive offers an effective strategy to mitigate these issues. The bulky organic guanidine cation preferentially adsorbs on the Zn anode surface, providing an electrostatic shielding effect to shield the “tip effect” and homogenize the local electric field. Simultaneously, the corresponding SO_4_^2–^ anion in ZT electrolyte triggers the in situ formation of a uniform and dense ZHS‐SEI layer on the Zn anode surface [34, 35]. This protective layer effectively isolates the anode from direct contact with the bulk electrolyte, thereby further mitigating side reactions and enhancing the stability and reversibility of the Zn anode.

**FIGURE 2:**
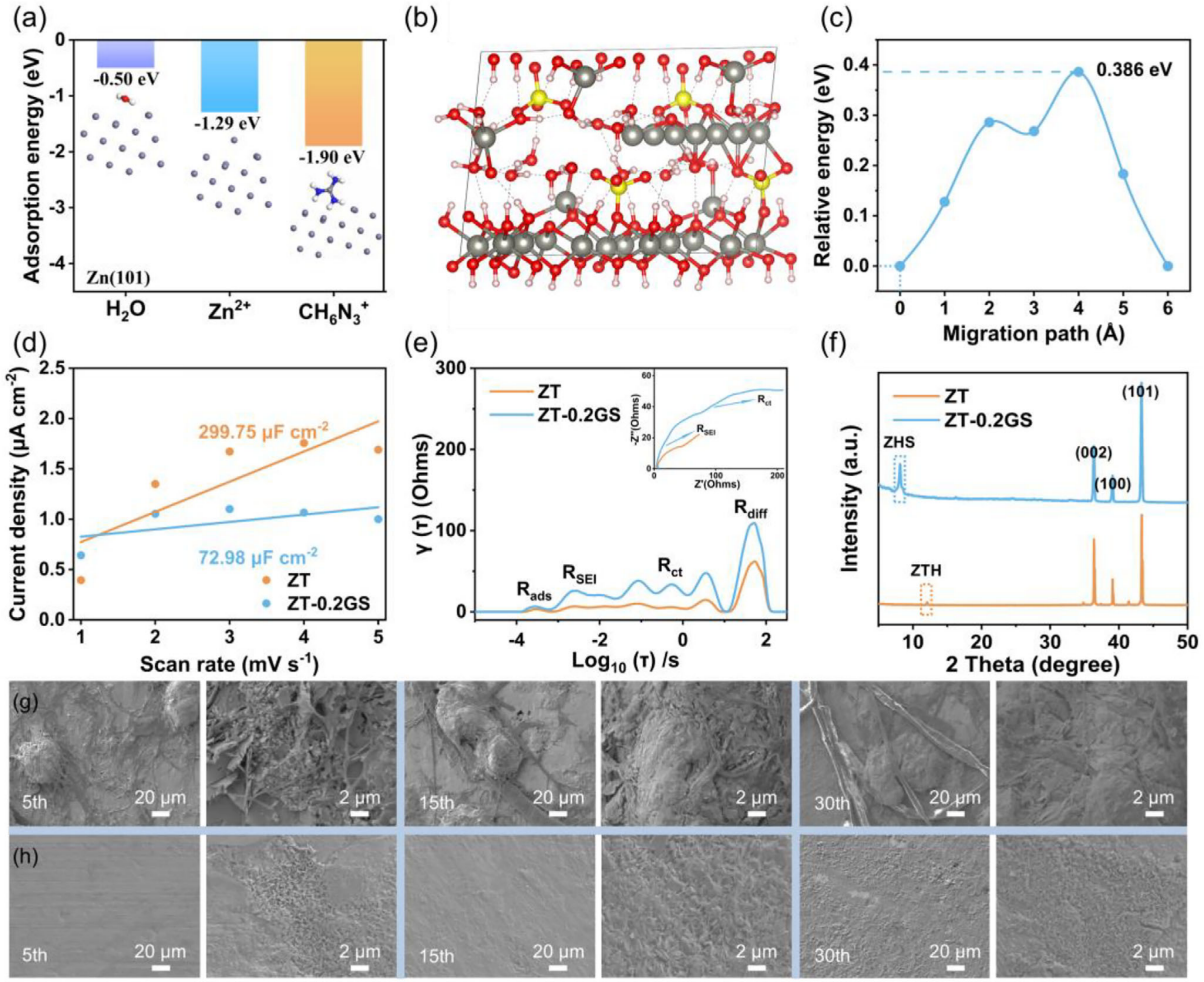
(a) Comparison of adsorption energy on the Zn (101) crystal plane. (b) DFT calculation for the typical inorganic ZHS. (c) Corresponding migration energy barriers within the ZHS structure. (d) Electric double‐layer capacitances in the ZT and ZT‐0.2GS electrolytes. (e) DRT analysis of Zn||Zn symmetric cells after 20 cycles, with the inset showing EIS impedance spectra. (f) XRD patterns of Zn anodes cycled for 20 cycles in ZT and ZT‐0.2GS electrolytes. (g,h) SEM images of Zn anodes after 5, 15, and 30 cycles in ZT and ZT‐0.2GS electrolytes.

The cation–anion synergistic mechanism of the GS additive was systematically investigated by theoretical calculations and experimental studies. As shown in Figure 2a, first‐principles calculations reveal that the adsorption energies of H_2_O molecules and Zn^2+^ on the Zn (101) surface are −0.5 and −1.29 eV, respectively (Table S1). Notably, CH_6_N_3_^+^ cations exhibit a significantly stronger adsorption energy of −1.90 eV, indicating the preferential adsorption onto the Zn anode surface, which helps shield the “tip effect” and promotes uniform Zn deposition [36]. The preferential adsorption of GS was further examined through EDL capacitance measurements (Figures 2d and S1). The calculated EDL capacitance for the baseline ZT electrolyte is 299.75 µF cm^−2^. With the addition of GS, the capacitance decreased markedly to 72.98 µF cm^−2^, indicating that the GS additive preferentially adsorbs onto the Zn surface and effectively regulates its EDL structure [37].

DFT calculations were used to investigate the mechanism of the additive in forming the SEI layer. Figure 2b shows the possible migration channel in the tunnel‐like framework of ZHS. The energy barrier for Zn^2+^ migration through ZHS is 0.386 eV, indicating that the ZHS layer facilitates rapid Zn^2+^ transport (Figure 2c). Electrochemical impedance spectroscopy (EIS) was conducted to support the formation of a robust interfacial layer. In the symmetric Zn||Zn cell with ZT‐0.2GS electrolyte, a pronounced resistance component attributed to the SEI (*R*_SEI_) appears in the high‐frequency region, confirming the in situ formation of a ZHS‐SEI protective layer on the Zn anode surface [38, 39]. Distribution of relaxation times (DRT) analysis is an effective technique to correlate relaxation times with characteristic electrochemical processes by deconvoluting EIS data. In the DRT plots, the ZT−0.2GS electrolyte exhibits a more prominent SEI‐related peak [40], which is consistent with the EIS results (Figure 2e). These findings are further corroborated by x‐ray diffraction (XRD) analysis of Zn anodes extracted after 20 cycles at 1 mA cm^−2^ and 1 mAh cm^−2^ (Figure 2f). The diffraction peak located at approximately 12.1° can be assigned to ZTH, indicating the occurrence of continuous side reactions in the ZT electrolyte. In contrast, the characteristic peak at approximately 8.1° observed in the ZT‐0.2GS electrolyte can be well indexed to ZHS, confirming the preferential formation of a ZHS‐rich interphase. Theoretically, Zn metal is thermodynamically unstable in both ZnSO_4_ (ZSO) and ZT electrolytes. The inevitable HER, accompanied by the increased local pH value, leads to the formation of ZHS or ZTH by‐products that are randomly distributed on the Zn surface and cannot effectively stabilize the Zn/electrolyte interface. Compared with the loosely distributed byproducts formed in the ZSO, ZSO‐GS, and ZT electrolyte, the ZHS phase formed in the ZT‐0.2GS electrolyte is more uniform and densely covered on the Zn anode surface by taking advantage of the complementary roles of ZT and SO_4_^2−^ (Figure S2). This ZHS‐SEI layer efficiently blocks the direct contact between electrolyte and the Zn anode, thereby suppressing parasitic reactions and the formation of ZTH. Furthermore, scanning electron microscopy (SEM) images of the Zn deposition after 5, 15, and 30 cycles at 1 mA cm^−2^ and 1 mAh cm^−2^ demonstrate that the GS additive facilitates smooth and uniform Zn deposition, eliminating dendrite growth (Figure 2g,h). These findings highlight the dual role of CH_6_N_3_^+^ and SO_4_^2–^ in modulating interfacial chemistry and improving interfacial stability.

**FIGURE 3:**
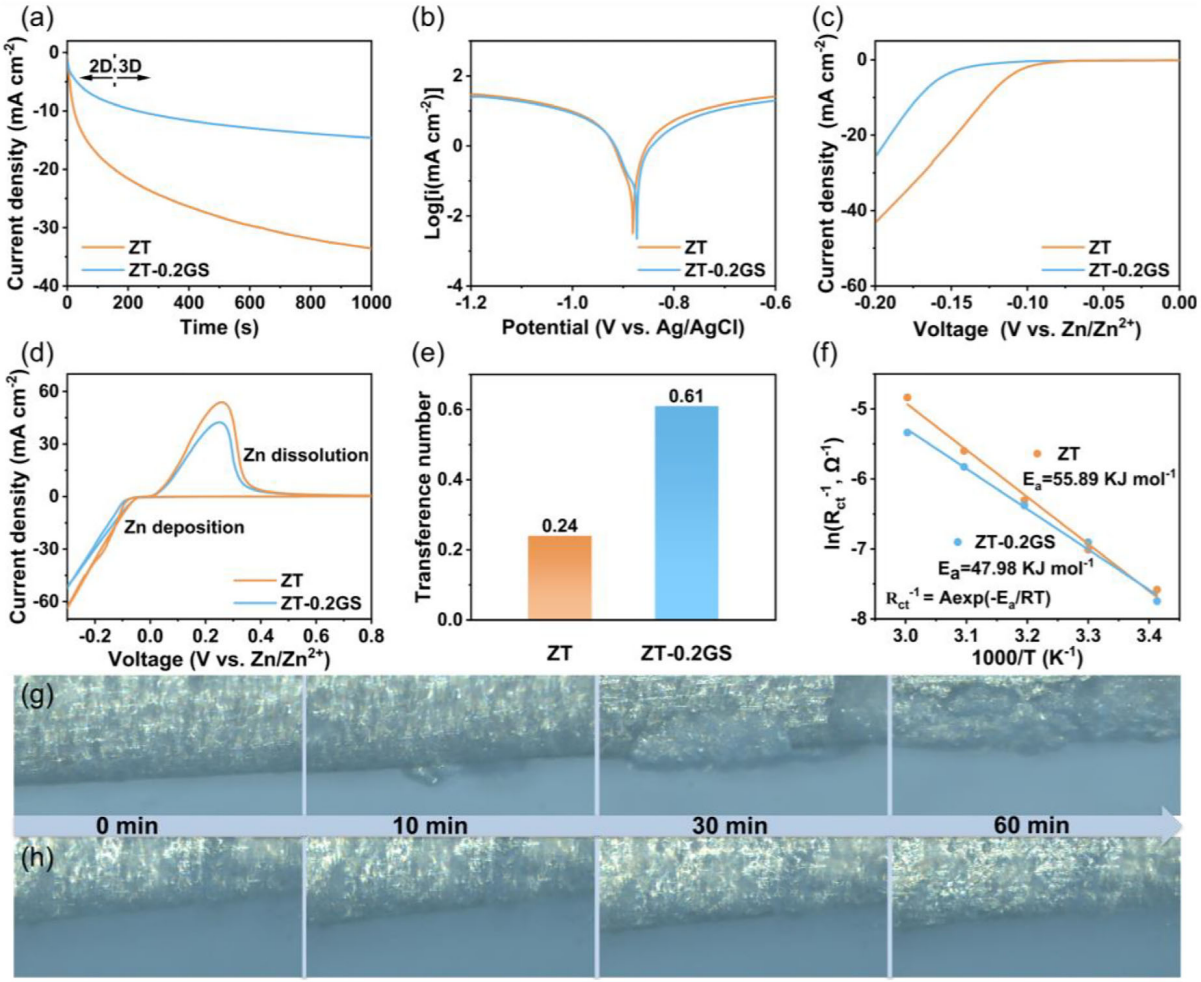
(a) Chronoamperometry curves of Zn^2+^ deposition behaviors. (b) Tafel curves and (c) LSV curves in various electrolytes. (d) The cyclic voltammetry test of Zn||Ti cells. (e) The Zn^2+^ transference number in the ZT and ZT‐0.2GS. (f) The Arrhenius curves and comparison of the activation energies. (g,h) In situ observation of Zn deposition process in ZT and ZT‐0.2GS electrolytes.

Building upon the cation–anion synergistic mechanism, the effect of the GS additive on Zn nucleation and growth behavior was further investigated. To investigate the nucleation process, chronoamperometry tests were conducted at a potential of −150 mV using Zn||Zn symmetric cells. As shown in Figure 3a, the current density in the ZT electrolyte continuously increased, which implies the uncontrolled 2D diffusion and uneven Zn deposition. In contrast, the introduction of the GS additive resulted in a rapid current stabilization, indicating a more uniform nucleation process and constrained 2D Zn^2+^ diffusion. The preferential adsorption of CH_6_N_3_^+^ cations on the Zn surface plays a critical role in homogenizing the electric field, thereby facilitating 3D Zn^2+^ diffusion. Benefiting from this behavior, uniform Zn deposition is predictable in ZT‐0.2GS electrolyte. To evaluate the corrosion resistance imparted by the GS additive, linear sweep voltammetry (LSV) was performed at a scan rate of 5 mV s^−1^ using Zn as the working electrode, a platinum mesh as the counter electrode, and an Ag/AgCl as the reference. As shown in Figure 3b, the ZT‐0.2GS exhibits a higher corrosion potential and a lower corrosion current, indicating enhanced corrosion resistance and improved Zn anode stability [41].

In addition, the LSV test of the Zn||Ti asymmetric cells confirmed that GS can effectively inhibit the HER (Figure 3c). Cyclic voltammetry (CV) tests at 0.5 mV s^−1^ were conducted to compare Zn^2+^ nucleation potentials in Zn||Ti cells using ZT and ZT‐0.2GS electrolytes. As shown in Figure 3d, the GS‐containing electrolyte exhibits reduced electrochemical polarization during Zn plating/stripping, suggesting improved interfacial compatibility and more reversible Zn electrochemistry [42]. The Zn^2+^ transference number is a critical parameter that affects the concentration polarization and the reversibility of the Zn anode. In ZT electrolyte, the Zn^2+^ transference number is only 0.24, while the introduction of GS additive significantly increases it to 0.61 (Figures 3e and S3). This substantial improvement contributes to more efficient Zn^2+^ transport and provides a further explanation for the reduced electrochemical polarization in GS‐containing electrolyte. To further assess Zn^2+^ kinetics, an EIS test was conducted over a temperature range of 293.15–333.15 K. According to the Arrhenius equation (1/*R*_ct_ = Aexp(−*E*_a_/RT), the activation energy (E_a_) for Zn^2+^ deposition was calculated. As depicted in Figures 3f and S4, ZT‐0.2GS electrolyte exhibits a lower *E*_a_ 47.98 KJ mol^−1^ compared to the ZT electrolyte (55.89 KJ mol^−1^), suggesting decreased energy barrier for Zn^2+^ desolvation kinetics [43, 44]. To visualize the Zn deposition behavior, a homemade Zn||Zn cell was constructed, allowing real‐time observation of the Zn anode cross‐section under an optical microscope. After 60 min of deposition, the Zn anode cycled in ZT‐0.2GS exhibits smooth and uniform growth without irregular accumulation (Figure 3h). In contrast, the anode in the ZT electrolyte shows highly uneven deposition with pronounced surface roughness and irregular accumulation (Figure 3g). These results collectively demonstrate that the GS additive not only enhances ion transport properties but also effectively modulates Zn^2+^ nucleation and growth, contributing to uniform and dendrite‐free Zn deposition.

**FIGURE 4:**
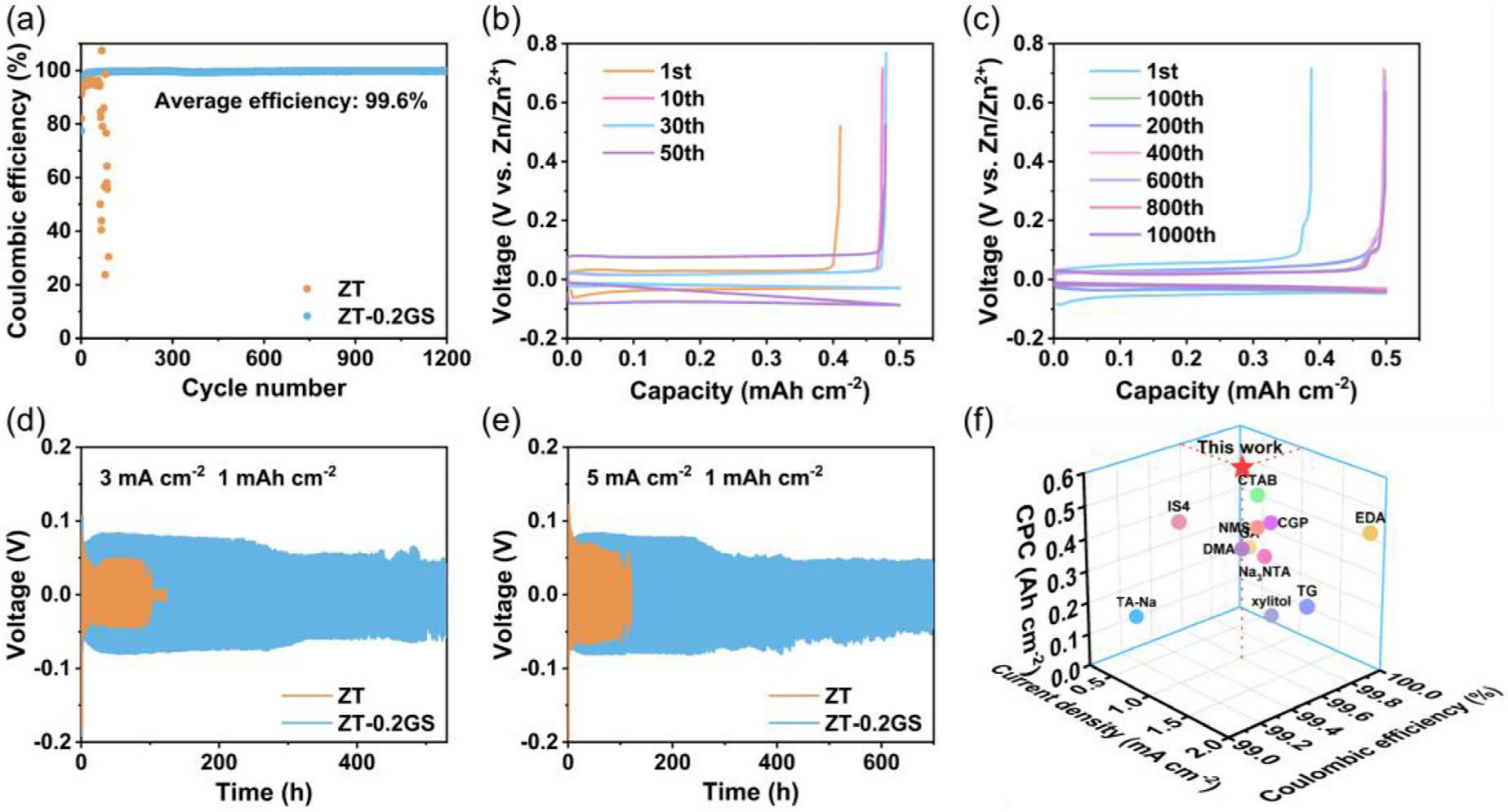
(a) Coulombic efficiency of the Zn||Cu cells using different electrolytes. (b,c) Corresponding voltage profiles of ZT and ZT‐0.2GS electrolytes. (d,e) Long‐term cycling performances of Zn||Zn cells under various current densities. (f) Comparison of cumulative plating capacity and Coulombic efficiency with recently reported works.

To gain insight into the reversibility and stability of Zn plating/stripping behavior, Zn||Cu and Zn||Zn cells were assembled and systematically tested. CE is an important index for assessing the reversibility of batteries [4]. The optimum concentration was obtained by comparing the CE of Zn||Cu cells with different GS concentrations. As shown in Figures 4a and S5, the ZT‐0.2GS electrolyte achieved an average CE of 99.6% over 1200 cycles at a current density of 1 mA cm^−2^ with a capacity of 0.5 mAh cm^−2^, corresponding to a cumulative plating capacity of 0.6 Ah cm^−2^. This result highlights the excellent reversibility and stability of Zn plating/stripping in the ZT‐0.2GS electrolyte. Additionally, the corresponding voltage‐capacity curves also show reduced voltage hysteresis in the electrolyte with GS additive (Figure 4b,c). Control experiments further reveal that neither the guanidinium cation nor the sulfate anion alone can achieve comparable electrochemical performance, confirming that the excellent reversibility and stability originate from their synergistic regulation of Zn deposition and interfacial chemistry (Figure S6). To evaluate the practical applicability of the electrolyte, Zn||Cu cells employing 10 µm Zn foils were assembled and tested at a high current density of 4 mA cm^−2^with an areal capacity of 4 mAh cm^−2^. Under these practically relevant conditions, the cell achieved a high Zn utilization of 68% and maintained stable cycling for over 50 cycles, highlighting the excellent reversibility and stability enabled by the GS additive (Figure S7). Long‐term cycling stability was further examined using Zn||Zn symmetric cells at current densities of 1, 3, and 5 mA cm^−2^. In Figures 4d,e and S8a, the symmetric cells with baseline ZT electrolyte exhibited poor cycling stability. In contrast, symmetric cells with ZT‐0.2GS electrolyte were able to cycle over 500 h at 1 and 3 mA cm^−2^, respectively. Remarkably, a long cycle life of more than 700 h can be achieved even at a higher current density of 5 mA cm^−2^. To further assess rate capability, Zn||Zn cells were tested at varying current densities from 1 to 5 mA cm^−2^. As indicated in Figure S8b, the ZT‐0.2GS electrolyte exhibits superior reversibility compared to the ZT electrolyte, demonstrating excellent performance across all conditions. According to CE parameter benchmarking from prior literature, ZT‐0.2GS electrolyte ranks among the top‐performance reported to date, validating its effectiveness in promoting high‐efficiency and long‐life AZIBs (Figure 4f and Table S2).

**FIGURE 5:**
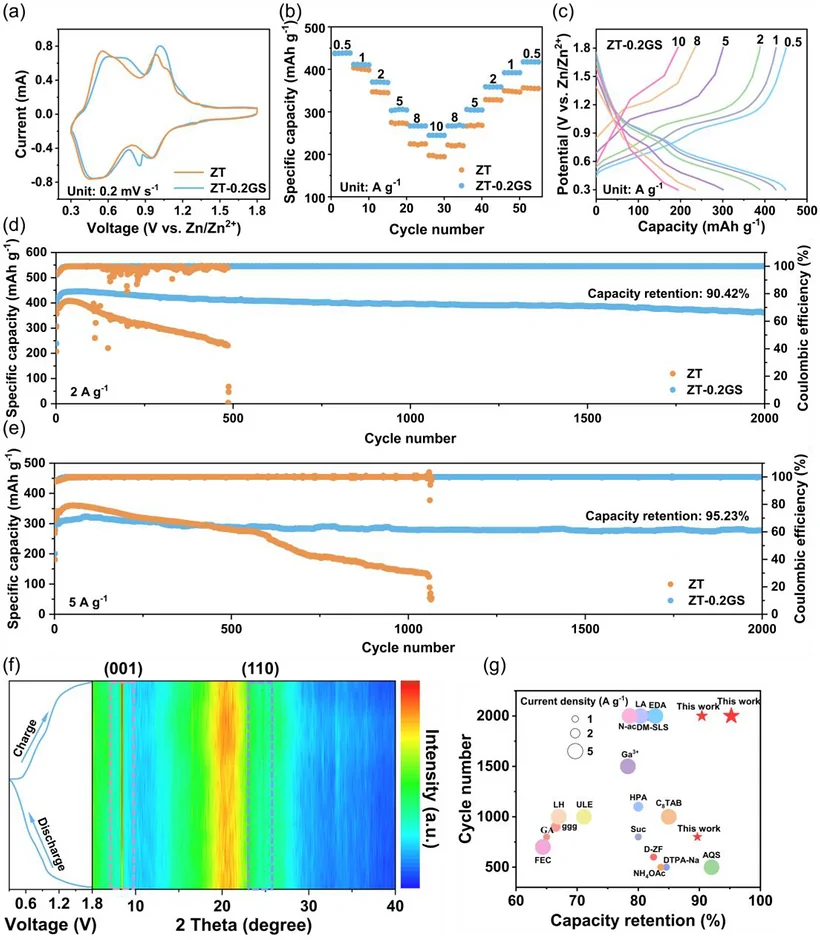
(a) CV curves of Zn||O_d_‐NVO·nH_2_O full cells in different electrolytes at 0.1 mV s^−1^. (b) Rate performance of Zn||O_d_‐NVO·nH_2_O full cells in different electrolytes at various current densities. (c) Corresponding voltage profiles in ZT‐0.2GS electrolyte. (d,e) Comparison of the cycling performances of Zn||O_d_‐NVO·nH_2_O full cells at 2 and 5 A g^−1^, respectively. (f) In situ XRD patterns of O_d_‐NVO·nH_2_O cathode in ZT‐0.2GS electrolyte. (g) Comparison of capacity retention with recently reported works.

To verify the practical applicability of the GS additive in full cells, an O_d_‐NVO·nH_2_O cathode was synthesized by a simple hydrothermal method, followed by coating and drying (Figure S9). Figure 5a shows the CV curves of Zn||O_d_‐NVO·nH_2_O full cells in different electrolytes. These two curves exhibit similar redox peaks, indicating that the GS additive does not alter the intrinsic charge storage mechanism of the cathode material [45]. The rate performance of the full cells was tested across a range of current densities from 0.5 to 10 A g^−1^ (Figure 5b). The capacity of the full cell in the ZT electrolyte significantly declined at higher current densities. On the contrary, the ZT‐0.2GS‐based full cells exhibited excellent rate performance, confirming enhanced reversibility and kinetics. Corresponding voltage profiles further prove the improved rate performance, reversibility, and kinetics (Figures 5c and S10). The long‐term cycling performances further highlight the practical applicability of the GS additive. Compared to the rapid capacity degradation observed in the full cell using ZT electrolyte, the ZT‐0.2GS electrolyte achieves a high‐capacity retention of 89.68% after 800 cycles at 1A g^−1^ (Figure S11). Remarkably, impressive capacity retentions of 90.42% and 95.23% can be achieved even after 2000 cycles in the ZT‐0.2GS electrolyte at current densities of 2 and 5 A g^−1^, respectively (Figure 5d,e). These results clearly demonstrate the practicability of the GS additive in enhancing the long‐term performance of AZIBs. To further evaluate the universality and practicability of the GS additive, additional validations were conducted under more practical conditions. High‐mass‐loading Zn||O_d_‐NVO·nH_2_O full cells (∼10 mg cm^−^^2^) employing the ZT‐0.2GS electrolyte maintained stable cycling for over 140 cycles at 1 A g^−^^1^, whereas the counterpart using the ZT electrolyte failed within 20 cycles (Figure S12). Furthermore, the electrolyte strategy was successfully extended to a fundamentally different Zn–I_2_ chemistry using a high iodine loading (∼10 mg cm^−^^2^) cathode and a thin Zn foil anode (10 µm), delivering a capacity retention of 80.99% after 500 cycles compared with only 36.27% for the baseline electrolyte (Figure S13). These results demonstrate the good generality and practical relevance of the proposed cation–anion synergistic strategy.

To elucidate the role of the GS additive in the structural evolution of O_d_‐NVO·nH_2_O cathode material during the charging and discharging processes, in‐situ XRD tests were conducted at a current density of 0.2 A g^−1^. As illustrated in Figures 5f and S14, the introduction of the GS additive ensures the structural integrity of the O_d_‐NVO·nH_2_O cathode material, as evidenced by the stable (001) and (110) diffraction peaks [46]. In addition, the GS additive can effectively suppress the formation of Zn_3_(OH)_2_V_2_O_7_·2H_2_O, thereby improving the reversibility and structural stability of the cathode (Figures S15 and S16) [47, 48]. The nearly overlapping diffraction peaks during the charging and discharging processes further demonstrate the reversibility and structure stability of the cathode in the ZT‐0.2GS electrolyte. Finally, as shown in Figure 5g and Table S3, a comparison with previously reported additives further highlights the effectiveness of GS in boosting AZIBs performance.

## 3 Conclusion

In summary, a novel electrolyte additive, guanidine sulfate, featuring a cation‐anion synergistic mechanism, has been demonstrated to effectively protect the Zn anode and enhance the overall performance of AZIBs. Both theoretical calculations and experimental results confirm that guanidinium cations preferentially adsorb onto the Zn anode surface, providing an electrostatic shielding effect that promotes uniform Zn^2+^ deposition and suppresses dendrite formation. Simultaneously, sulfate anions contribute to the formation of the in situ ZHS‐SEI, preventing direct contact between the Zn anode and the electrolyte, thereby mitigating parasitic reactions such as hydrogen evolution and corrosion. Leveraging the synergistic effect of cations and anions, significant suppression of water‐induced side reactions and enhancement of reversibility and stability can be realized. As a result, Zn||Zn symmetric cells incorporating guanidine sulfate additive exhibited outstanding cycling stability for over 700 h at 5 mA cm^−2^. Zn||O_d_‐NVO·nH_2_O full cells retained over 90% of their capacity after 2000 cycles at high current densities of 2 and 5 A g^−1^, confirming the additive's efficacy in practical applications. This work highlights the critical role of cation−anion synergy in electrolyte design and offers new insights into the development of advanced AZIBs with improved stability and reversibility.

## Conflicts of Interest

The authors declare no conflicts of interest.

## Supporting information




**Supporting File**: anie73458‐sup‐0001‐SuppMat.docx.

## Data Availability

The data that support the findings of this study are available from the corresponding author upon reasonable request.

## References

[1] X. Guo , C. Li , Y. Zhou , Y. Chen , W. Deng , and R. Li , “Ti_2_ O_3_ (H_2_ PO_4_ )_2_ 2H_2_ O as a Novel Intercalated Anode for Ultralong Lifespan “Rocking‐Chair” Aqueous Zinc‐Ion Batteries,” Angewandte Chemie International Edition 64 (2025): e202502446, 10.1002/anie.202502446. 40097921

[2] H. Chen , W. Huang , Z. Deng , et al., “Advancements in Zinc Reversibility and Utilization for Practical Aqueous Zinc‐Ion Battery Applications,” Advanced Energy Materials 15 (2025): 2501052, 10.1002/aenm.202501052.

[3] Y. Liu , L. Lin , T. Zhang , et al., “A Cyano Cobalt “Electron Transfer Bridge” Boosting the Two‐Electron Reaction of a MnO_2_ Cathode With Long Lifespan in Aqueous Zinc Batteries,” Energy & Environmental Science 17 (2024): 2521–2529, 10.1039/D3EE03711H.

[4] R. Chen , C. Zhang , J. Li , et al., “A Hydrated Deep Eutectic Electrolyte With Finely‐Tuned Solvation Chemistry for High‐Performance Zinc‐Ion Batteries,” Energy & Environmental Science 16 (2023): 2540–2549, 10.1039/D3EE00462G.

[5] X. Zhou , B. Wen , Y. Cai , et al., “Interfacial Engineering for Oriented Crystal Growth Toward Dendrite‐Free Zn Anode for Aqueous Zinc Metal Battery,” Angewandte Chemie International Edition 63 (2024): e202402342, 10.1002/anie.202402342. 38491787

[6] J. Wei , P. Zhang , J. Sun , et al., “Advanced Electrolytes for High‐Performance Aqueous Zinc‐Ion Batteries,” Chemical Society Reviews 53 (2024): 10335–10369, 10.1039/D4CS00584H. 39253782

[7] J. Wang , H. Zhang , L. Yang , S. Zhang , X. Han , and W. Hu , “In Situ Implanting 3D Carbon Network Reinforced Zinc Composite by Powder Metallurgy for Highly Reversible Zn‐Based Battery Anodes,” Angewandte Chemie International Edition 63 (2024): e202318149, 10.1002/anie.202318149. 38169516

[8] Q. Wu , J. Huang , J. Zhang , et al., “Multifunctional Cellulose Nanocrystals Electrolyte Additive Enable Ultrahigh‐Rate and Dendrite‐Free Zn Anodes for Rechargeable Aqueous Zinc Batteries,” Angewandte Chemie International Edition 63 (2024): e202319051, 10.1002/anie.202319051. 38305690

[9] R. Chen , Y. Zhong , P. Jiang , et al., “Untangling the Role of Capping Agents in Manipulating Electrochemical Behaviors Toward Practical Aqueous Zinc‐Ion Batteries,” Advanced Materials 37 (2025): 2412790, 10.1002/adma.202412790. 39777795 PMC12631510

[10] D. Xu , X. Ren , H. Li , et al., “Chelating Additive Regulating Zn‐Ion Solvation Chemistry for Highly Efficient Aqueous Zinc‐Metal Battery,” Angewandte Chemie International Edition 63 (2024): e202402833, 10.1002/anie.202402833. 38535776

[11] Z. Zhang , P. Wang , C. Wei , J. Feng , S. Xiong , and B. Xi , “Synchronous Regulation of D–Band Centers in Zn Substrates and Weakening Pauli Repulsion of Zn Ions Using the Ascorbic Acid Additive for Reversible Zinc Anodes,” Angewandte Chemie International Edition 63 (2024): e202402069, 10.1002/anie.202402069. 38466145

[12] L. Lin , Z. Shao , S. Liu , et al., “High‐Entropy Aqueous Electrolyte Induced Formation of Water‐Poor Zn^2+^ Solvation Structures and Gradient Solid‐Electrolyte Interphase for Long‐Life Zn‐Metal Anodes,” Angewandte Chemie International Edition 64 (2025): e202425008, 10.1002/anie.202425008. 39847035

[13] M. Liu , Y. Wang , Y. Li , et al., “Dual‐Functional Interfacial Layer Enabled by Gating‐Shielding Effects for Ultra‐Stable Zn Anode,” Advanced Materials 36 (2024): 2406145, 10.1002/adma.202406145. 39221543

[14] Y. Li , B. Ping , J. Qu , et al., “Constructing Autoregulative Electric Double Layer through Dielectric Effect Toward Fast Charging Zinc Metal Anode,” Advanced Energy Materials 15 (2025): 2405804, 10.1002/aenm.202405804.

[15] L. Liu , X. Wang , Z. Hu , et al., “Electric Double Layer Regulator Design Through a Functional Group Assembly Strategy Towards Long‐Lasting Zinc Metal Batteries,” Angewandte Chemie International Edition 63 (2024): e202405209, 10.1002/anie.202405209. 38712643

[16] Y. Huang , H. Yan , W. Liu , and F. Kang , “Transforming Zinc‐Ion Batteries With DTPA‐Na: A Synergistic SEI and CEI Engineering Approach for Exceptional Cycling Stability and Self‐Discharge Inhibition,” Angewandte Chemie International Edition 63 (2024): e202409642, 10.1002/anie.202409642. 39037894

[17] R. Chen , W. Zhang , C. Guan , et al., “Rational Design of an In‐Situ Polymer‐Inorganic Hybrid Solid Electrolyte Interphase for Realising Stable Zn Metal Anode Under Harsh Conditions,” Angewandte Chemie International Edition 63 (2024): e202401987, 10.1002/anie.202401987. 38526053 PMC11497294

[18] P. Xu , M. Xu , J. Zhang , et al., “In‐Situ Solid Electrolyte Interface via Dual Reaction Strategy for Highly Reversible Zinc Anode,” Angewandte Chemie International Edition 63 (2024): e202407909, 10.1002/anie.202407909. 38993054

[19] M. Cui , L. Yu , J. Hu , S. He , C. Zhi , and Y. Huang , “Tailored Polymer‐Inorganic Bilayer SEI With Proton Holder Feature for Aqueous Zn Metal Batteries,” Angewandte Chemie International Edition 64 (2025): e202423531, 10.1002/anie.202423531. 39811983

[20] K. Ouyang , S. Chen , W. Ling , et al., “Synergistic Modulation of In‐Situ Hybrid Interface Construction and pH Buffering Enabled Ultra‐Stable Zinc Anode at High Current Density and Areal Capacity,” Angewandte Chemie International Edition 62 (2023): e202311988, 10.1002/anie.202311988. 37743256

[21] X. Guo , K. Zhang , D. Han , et al., “A Spatiotemporal‐Orchestrated Hybrid Interphase for Highly Reversible Zinc Batteries,” Advanced Energy Materials 15 (2025): 2501180, 10.1002/aenm.202501180.

[22] G. Ma , W. Yuan , X. Li , et al., “Organic Cations Texture Zinc Metal Anodes for Deep Cycling Aqueous Zinc Batteries,” Advanced Materials 36 (2024): 2408287, 10.1002/adma.202408287. 38967293

[23] X. Yang , Q. Zhou , S. Wei , et al., “Anion Additive Integrated Electric Double Layer and Solvation Shell for Aqueous Zinc Ion Battery,” Small Methods 8 (2024): 2301115, 10.1002/smtd.202301115. 38145365

[24] R. Yao , L. Qian , Y. Sui , et al., “A Versatile Cation Additive Enabled Highly Reversible Zinc Metal Anode,” Advanced Energy Materials 12 (2022): 2102780, 10.1002/aenm.202102780.

[25] X. Zhang , L. Chen , R. Orenstein , et al., “Zincophilic and Hydrophobic Groups of Surfactant‐Type Electrolyte Additive Enabled Stable Anode/Electrolyte Interface Toward Long‐Lifespan Aqueous Zinc Ion Batteries,” Energy Storage Materials 70 (2024): 103500, 10.1016/j.ensm.2024.103500.

[26] M. Yang , J. Zhu , S. Bi , et al., “The Construction of Anion‐Induced Solvation Structures in Low‐Concentration Electrolyte for Stable Zinc Anodes,” Angewandte Chemie International Edition 63 (2024): e202400337, 10.1002/anie.202400337. 38351433

[27] G. Zeng , Q. Sun , S. Horta , et al., “Modulating the Solvation Structure to Enhance Amorphous Solid Electrolyte Interface Formation for Ultra‐Stable Aqueous Zinc Anode,” Energy & Environmental Science 18 (2025): 1683–1695, 10.1039/D4EE03750B.

[28] T. Sun , S. Zheng , H. Du , and Z. Tao , “Synergistic Effect of Cation and Anion for Low‐Temperature Aqueous Zinc‐Ion Battery,” Nano‐Micro Letters 13 (2021): 204, 10.1007/s40820-021-00733-0. 34625857 PMC8501177

[29] C. Xu , H. Wang , C. Lei , J. Li , W. Ma , and X. Liang , “Fast Single Metal Cation Conduction in Ion‐Water Aggregated Aqueous Battery Electrolytes,” Nature Communications 16 (2025): 4574, 10.1038/s41467-025-59958-x. PMC12084611 40379654

[30] I. Al Kathemi , Z. Slim , F. Igoa Saldaña , et al., “Tailoring the Solvation of Aqueous Zinc Electrolytes by Balancing Kosmotropic and Chaotropic Ions,” ACS Nano 19 (2025): 6388–6398, 10.1021/acsnano.4c16521. 39915274 PMC11841044

[31] Y. Xu , K. Chen , M. Xu , et al., “Zinc Chemistry Regulated By Chitosan‐Based Poly(Aprotic/Protic Ionic Liquid)s With Multi‐Anion–Cation Interactions for Highly Reversible Zn‐Ion Batteries,” Energy & Environmental Science 18 (2025): 1560–1571, 10.1039/D4EE05442C.

[32] Z. Zhang , X. Lan , G. Liao , et al., “Coupling Zn^2+^ Ferrying Effect With Anion–π Interaction to Mitigate Space Charge Layer Enables Ultra‐High Utilization Rate Zn Anode,” Angewandte Chemie International Edition 64 (2025): e202503396, 10.1002/anie.202503396. 40119778

[33] F. Wu , J. Zhang , L. Ma , et al., “Directing Zn Growth With Biased Adsorption of Straight‐Chain Molecules for Superior Zn Anode Stability,” Angewandte Chemie International Edition 64 (2025): e202421787, 10.1002/anie.202421787. 39659171

[34] Y. Lv , C. Huang , M. Zhao , et al., “Synergistic Anion–Cation Chemistry Enables Highly Stable Zn Metal Anodes,” Journal of the American Chemical Society 147 (2025): 8523–8533, 10.1021/jacs.4c16932. 40033817

[35] Q. Wang , Z. Zhang , Z. Hu , et al., “Manipulating Interphase Chemistry for Aqueous Zn Stabilization: The Role of Supersaturation,” Angewandte Chemie International Edition 64 (2025): e202420772, 10.1002/anie.202420772. 39731396

[36] J. Cao , Y. Jin , H. Wu , et al., “Enhancing Zinc Anode Stability With Gallium Ion‐Induced Electrostatic Shielding and Oriented Plating,” Advanced Energy Materials 15 (2025): 2403175, 10.1002/aenm.202403175.

[37] N. Hu , W. Lv , W. Chen , et al., “A Double‐Charged Organic Molecule Additive to Customize Electric Double Layer for Super‐Stable and Deep‐Rechargeable Zn Metal Pouch Batteries,” Advanced Functional Materials 34 (2024): 2311773, 10.1002/adfm.202311773.

[38] R. Chen , W. Zhang , Q. Huang , et al., “Trace Amounts of Triple‐Functional Additives Enable Reversible Aqueous Zinc‐Ion Batteries From a Comprehensive Perspective,” Nano‐Micro Lett 15 (2023): 81, 10.1007/s40820-023-01050-4. PMC10066055 37002511

[39] Y. Chu , S. Zhang , S. Wu , Z. Hu , G. Cui , and J. Luo , “In Situ Built Interphase With High Interface Energy and Fast Kinetics for High Performance Zn Metal Anodes,” Energy & Environmental Science 14 (2021): 3609–3620, 10.1039/D1EE00308A.

[40] M. Liu , K. Yang , Q. Xie , et al., “Operando Evolution of a Hybrid Metallic Alloy Interphase for Reversible Aqueous Zinc Batteries,” Angewandte Chemie International Edition 64 (2025): e202416047, 10.1002/anie.202416047. 39671251 PMC11773305

[41] K. Liu , M. Sun , Y. Wu , et al., “Binary Electrolyte Additive‐Reinforced Interfacial Molecule Adsorption Layer for Ultra‐Stable Zinc Metal Anodes,” Advanced Materials 37 (2025): 2420079, 10.1002/adma.202420079. 40109192 PMC12051903

[42] M. Luo , X. Gan , C. Zhang , et al., “Overcoming Obstacles in Zn‐Ion Batteries Development: Application of Conductive Redox‐Active Polypyrrole/Tiron Anolyte Interphase,” Advanced Functional Materials 33 (2023): 2305041, 10.1002/adfm.202305041.

[43] B. Wu , T. Yan , S. Liu , et al., “Reconstructing Electric Double Layer With β‐diketone Additive for Highly Invertible Zn Anode,” Advanced Functional Materials 35 (2025): 2421244, 10.1002/adfm.202421244.

[44] X. Chen , S. Li , K. Wang , H. Zhao , G. He , and Y. Bai , “Halogenated Solvation Structure and Preferred Zn (002) Deposition via Trace Additive Towards High Reversibility for Aqueous Zinc‐Ion Batteries,” Energy Storage Mater 73 (2024): 103869, 10.1016/j.ensm.2024.103869.

[45] Z. Luo , Y. Xia , S. Chen , et al., “A Homogeneous Plating/Stripping Mode With Fine Grains for Highly Reversible Zn Anodes,” Energy & Environmental Science 17 (2024): 6787–6798, 10.1039/D4EE02264E.

[46] M. Ni , M. Qin , H. Chang , et al., “Cations‐Pillared and Polyaniline‐Encapsulated Vanadate Cathode for High‐Performance Aqueous Zinc‐Ion Batteries,” Chemsuschem 17 (2024): e202400526, 10.1002/cssc.202400526. 38679575

[47] X. Wang , Y. Wang , Y. Jiang , et al., “Tailoring Ultrahigh Energy Density and Stable Dendrite‐Free Flexible Anode With Ti_3_C_2_T* _x_ * MXene Nanosheets and Hydrated Ammonium Vanadate Nanobelts for Aqueous Rocking‐Chair Zinc Ion Batteries,” Advanced Functional Materials 31 (2021): 2103210, 10.1002/adfm.202103210.

[48] L. Chen , H. Yue , Z. Zhang , et al., “Pre‐Removing Partial Ammonium Ions From the Interlayer of Ammonium Vanadate With Acid Treating for Quasi‐Solid‐State Flexible Zinc Ion Batteries,” Chemical Engineering Journal 455 (2023): 140679, 10.1016/j.cej.2022.140679.

