# Supplementary material for: Harnessing Cation–Anion Synergistic Effect for High‐Performance Aqueous Zinc‐Ion Batteries
Source: Angew Chem Int Ed Engl. 2026 Jul 2;65(36):e3172435. doi: 10.1002/anie.3172435 (PMC13528532; doi:10.1002/anie.3172435)
Supplement: Supplementary file 1 [file ANIE-65-e3172435-s001.docx]

**Supporting Information**

**Harnessing** **Cation-Anion Synergistic Effect for High-Performance Aqueous Zinc-Ion Batteries**

Weichen Li^+^, Jiyang Liu^+^, Junhong Guo, Anyao Song, Suli Chen, Ruwei Chen*, Yunpeng Zhong, Yongkang Xing, Jihao Sun, Zhihong Tian*, and Guanjie He*

W. Li, A. Song, Prof. Z. Tian

Engineering Research Center for Nanomaterials, Henan University, Kaifeng 475004, P. R. China

E-mail: [zhihong.tian@henu.edu.cn](mailto:zhihong.tian@henu.edu.cn)

J. Liu, Dr. R. Chen, Y. Zhong, Y. Xing, J. Sun, Prof. G. He

Department of Chemistry, University College London, London, WC1H 0AJ, UK
E-mail: ruwei.chen@ucl.ac.uk; g.he@ucl.ac.uk

J. Guo, Prof. S. Chen

Key Laboratory of Synthetic and Biological Colloids, Ministry of Education, School of Chemical and Material Engineering, Jiangnan University, Wuxi 214122, P. R. China

[+] These authors contributed equally to this work.

**Experimental Section**

**Material and Methods:** Zn (CF_3_SO_3_)_2_ (≥98%), I_2_ (≥99.8%), C_2_H_6_F_3_N_3_O_3_S, Ketjen black were purchased from Shanghai Macklin Biochemical Co., Ltd. Guanidine sulfate (≥99%), NH_4_VO_3_ (≥99%), Oxalic acid (≥98%), Ammonium fluoride (≥99.99%), ZnSO_4_·7H_2_O (AR), polyvinylidene fluoride, Super-P, polytetrafluoroethylene, carboxymethyl cellulose were purchased from Shanghai Aladdin Biochemical Technology Co., LTD. Zn foils (0.2 mm, ≥99.9%) were purchased from Sinopharm Group Chemical Reagent Co., LTD. Zinc foils (0.1 mm, ≥99.9% and 0.01 mm, ≥99.9%) were purchased from Shenzhen Kejing Technology Co., Ltd. Cu foil (≥99.9%) was purchased from Shenzhen Kejing Technology Co., LTD. Carbon paper was purchased from Toray Industries, Japan. Glass-fiber (GF/D) was purchased from Whatman company, The United Kingdom. YP-80F was purchased from Kuraray Co., Ltd., Japan.

For cell assembly, the electrode sizes were specified as follows. The Zn foil and Cu foil used in Zn||Zn symmetric cells and Zn||Cu half-cells had diameters of 12 mm and 16.2 mm, respectively. For the Zn||O_d_‑NVO·nH_2_O full cells, Zn foil with a diameter of 16.2 mm was employed as the anode, while the O_d_‑NVO·nH_2_O cathode coated on carbon paper was punched into discs with a diameter of 12 mm.

**Electrolyte preparation:** A 2 mol·L^-1^ Zn (CF_3_SO_3_)_2_ aqueous solution was prepared as the baseline electrolyte (denoted as ZT). The designed electrolytes were prepared by adding various concentrations of GS additive (0.15, 0.2, and 0.3 mol·L^-1^) into the ZT electrolyte under 25 ℃ (denoted as ZT-0.15GS, ZT-0.2GS, ZT-0.3GS, respectively). The optimized electrolyte is ZT-0.2GS.

**Electrode preparation:** O_d_-NVO·nH_2_O cathode material was prepared by a hydrothermal method. Briefly, 5 mmol of ammonium metavanadate was dissolved in 30 mL deionized water under stirring for 10 mins. Subsequently, 8 mmol of anhydrous oxalic acid and 1 mmol of ammonium fluoride were added, and the mixture was continuously stirred for an additional 30 min. The resulting solution was transferred into a 50 mL Teflon-lined stainless-steel autoclave and heated at 180 °C for 6 h. After naturally cooling to room temperature, the obtained product was collected by centrifugation, washed several times with deionized water, and freeze-dried for 48 h to obtain O_d_-NVO·nH_2_O. ^[S27]^

For the cathode preparation, O_d_-NVO·nH_2_O, Super-P, and polyvinylidene fluoride were mixed at a mass ratio of 7:2:1. N-Methyl-2-pyrrolidone was then added dropwise, and the mixture was thoroughly ground to form a homogeneous slurry. The slurry was uniformly coated onto hydrophilic carbon paper and dried in a vacuum oven at 60 °C for 12 h. The areal mass loading of active material was controlled at approximately 1.5 mg cm⁻². For the in situ XRD measurements, 250-mesh stainless-steel mesh rather than carbon paper was employed as the current collector to avoid diffraction interference from carbon materials. The corresponding active material loading was increased to approximately 3.5 mg cm⁻² to enhance the diffraction signal intensity. For high-loading cathodes, O_d_-NVO·nH_2_O, Ketjen black, and polytetrafluoroethylene binder were mixed at a mass ratio of 7:2:1 and then pressed onto 100-mesh titanium mesh. The electrode was subsequently dried naturally at room temperature. The areal mass loading of active material was controlled at approximately 10 mg cm⁻².

Preparation of AC@I₂ Cathodes. AC@I₂ was synthesized using a sublimation-assisted method. Briefly, YP-80F activated carbon and iodine were mixed at a mass ratio of 1:1 and thoroughly ground in an agate mortar to obtain a homogeneous mixture. The mixture was transferred into a 50 mL Teflon-lined autoclave and heated at 120 °C for 6 h. After naturally cooling to room temperature, the AC@I₂ cathode material was collected. For electrode fabrication, AC@I₂, Ketjen black, and sodium carboxymethyl cellulose binder were mixed at a mass ratio of 8:1:1. Deionized water was added as a dispersant, and the mixture was ground until a homogeneous slurry was obtained. The slurry was uniformly coated onto carbon paper and dried at room temperature. The areal loading of iodine-containing active material was controlled at approximately 10 mg cm⁻².

**Materials characterizations:** The ex-situ and in-situ X-ray diffraction (XRD) patterns were obtained on a Bruker D8 ADVANCE with Cu Kα radiation. Scanning electron microscopy (SEM) was conducted on Gemini SEM 500. The in-situ optical microscope was conducted on Leica DM 6 M instrument.

Electrochemical tests: The electrochemical performances of Zn||Zn symmetric batteries, Zn||Cu asymmetric batteries, Zn||O_d_-NVO·nH_2_O full batteries were tested using CR 2032-coin cells. Galvanostatic discharge-charge process was conducted on a Neware battery testing system. The tests of cyclic voltammetry (CV), linear sweep voltammetry (LSV), chronoamperometry measurement (CA), and electrochemical impedance spectroscopy (EIS) were achieved by a CHI-660E electrochemical workstation. The calculation of activation energy is based on Arrhenius equation 1:

$\text{1/ }\text{R}_{\text{ct}}\text{=}\text{ }\text{A}\text{ }\text{exp(-}\frac{\text{E}_{\text{a}}}{\text{RT}}\text{)}$

Where, R_ct_ is the charge-transfer resistance, A is the frequency factor, and E_a_ is the activation energy.

The transference number of Zn^2+^ was calculated by the following equation 2:

$$\text{t}_{\text{Zn}^{\text{2+}}}\text{=}\text{ }\frac{\text{I}_{\text{S}}\left( \text{ ΔV-}\text{I}_{\text{0}}\text{R}_{\text{0}} \right)}{\text{I}_{\text{0}}\left( \text{ΔV-}\text{I}_{\text{s}}\text{R}_{\text{s}} \right)}$$

Where, I_0_ and R_0_ represent the initial current and initial resistance before polarization, I_S_ and R_S_ represent the steady-state current and steady-state resistance after polarization, and ΔV denotes the applied polarization voltage of 30 mV.

The Electric Double Layer Capacitance (EDLC) was calculated through the following Equation 3: $\text{C}_{\text{d}}\text{= }\frac{\text{i}_{\text{c}}}{\text{v}}$

Where, i_c_ represent the capacitive currents, *v* refers to the scan rate. The variable-rate CV scanning was performed on a Zn symmetric cell under the condition of -15 mV to 15 mV.

Computational method: The adsorption energy for H_2_O molecule, Zn^2+^, and CH_6_N_3_^+^ adsorption on Zn (101) surfaces was respectively calculated using the DMol3 module in Material Studio (MS) 2020. The adsorption energies E_ads_ was calculated according to the following Equation 4:

$$\text{E}_{\text{ads}}\text{=}\text{E}_{\text{total}}\text{-}\text{E}_{\text{adsorbate}}\text{-}\text{E}_{\text{Zn }\left( \text{101} \right)}$$

Where E_total_ is the total energy of the adsorption system, E_adsorbate_ and E_Zn (101)_ are the energies of the isolated adsorbate, H_2_O, CH_6_N_3_^+^ and Zn (101) substrate, respectively. The nudged-elastic band (NEB) method is employed to investigate the Zn diffusion barrier in the system. ^[S28]^

Figure S1 CV curves of Zn||Zn symmetric cell under various scanning rates.

Figure S2 (a) Coulombic efficiency of the Zn||Cu cells in ZSO-based electrolytes. (b) Long-term cycling performances of Zn||Zn cells in ZSO-based electrolytes. (c) XRD patterns of Zn anodes after prolonged cycling in ZSO-based electrolytes. (d-e) SEM images of Zn anodes after 5, 15, and 30 cycles in ZSO and ZSO-GS electrolytes, respectively.

Figure S3 CA tests and Electrochemical impedance spectroscopy (EIS) curves of the Zn||Zn symmetric cells in ZT and ZT-0.2GS electrolytes.

Figure S4 EIS curves of Zn||Zn symmetric cells under different temperatures. (a) ZT, (b) ZT-0.2GS.

Figure S5 CE comparison of Zn||Cu cells in (a) ZT-0.15GS, (b) ZT-0.3GS.

Figure S6 (a) Coulombic efficiencies of Zn||Cu cells at 1 mA cm^-2^ and 0.5 mAh cm^-2^. (b) Long-term cycling performance of Zn||Zn cells at 1 mA cm^-2^ and 1 mAh cm^-2^. (c) XRD results of Zn deposition after 5, 15 and 30 cycles. (d-e) SEM images of Zn deposition after 5, 15, and 30 cycles in ZT-ZSO and ZT-CH_6_N_3_^+^ electrolytes, respectively.

Figure S7 Coulombic efficiency of Zn||Cu cells employing 10 μm-thick Zn foils at a Zn depth of discharge (DOD) of 68%.

Figure S8 (a) Long-term Zn stripping/plating test. (b) Rate performance under different areal current densities and areal capacities.

Figure S9 X-ray diffraction pattern and scanning electron microscopy image of O_d_-NVO·nH_2_O cathode material.

Figure S10 Corresponding voltage profiles of Zn||O_d_-NVO·nH_2_O full cell in ZT electrolyte.

Figure S11 Comparison of the cycling performances of Zn||O_d_-NVO·nH_2_O full cells at 1A g^-1^.

Figure S12 (a) Long-term cycling performance of high mass loading Zn||O_d_-NVO·nH_2_O full cells. (b) Photograph of the high mass loading O_d_-NVO·nH_2_O cathode.

Figure S13 Long-term cycling performance of high-mass-loading Zn||AC@I_2_ full cells employing thin Zn foil anodes.

Figure S14 In situ XRD spectra of O_d_-NVO·nH_2_O cathode in ZT electrolyte.

Figure S15 (a) Corresponding XRD patterns of O_d_-NVO·nH_2_O cathode in ZT electrolyte, (b)

Corresponding XRD peak of the by-product.

Figure S16 Corresponding XRD patterns of O_d_-NVO·nH_2_O cathode in in ZT-0.2GS electrolyte.

**Table S1 Adsorption energy through Density functional theory (DFT) calculations.**

| Zn (101) substrate | E_total_ (kcal/mol) | E_Zn (101)_ (kcal/mol) | E_adsorbate_ (kcal/mol) | E_ads_ (kcal/mol) |
| --- | --- | --- | --- | --- |
| H_2_O-Zn | -32.902380 | -32.058439 | -0.342598 | -0.50134 |
| Zn^2+^-Zn | -44.352845 | -32.016536 | -11.044488 | -1.29182 |
| CH_6_N_3_^+^-Zn | -304.018312 | -32.109083 | -270.011471 | -1.897758 |

**Table S2 Comparison of CE and CPC of Zn//Cu cells with previous works concerning electrolyte additives.**

| Additive | Current density/Areal capacity (mA cm^-2^/mAh cm^-2^) | Cumulative plating capacity (mAh cm^-2^) | Coulombic efficiency  (%) | Reference |
| --- | --- | --- | --- | --- |
| TA-Na | 0.2/0.1 | 0.1 | 99.3 | [S1] |
| GA | 0.5/0.5 | 0.25 | 99.91 | [S2] |
| xylitol | 1/1 | 0.1 | 99.8 | [S3] |
| Na_3_NTA | 1/0.5 | 0.3 | 99.75 | [S4] |
| DMA | 1/0.5 | 0.35 | 99.6 | [S5] |
| CGP | 1/0.5 | 0.4 | 99.79 | [S6] |
| NMS | 1/0.5 | 0.4 | 99.7 | [S7] |
| CTAB | 1/0.5 | 0.5 | 99.7 | [S8] |
| IS4 | 1/0.5 | 0.5 | 99.2 | [S9] |
| TG | 2/1 | 0.3 | 99.49 | [S10] |
| EDA | 2/1 | 0.45 | 99.9 | [S11] |
| GS | 1/0.5 | 0.6 | 99.6 | **This work** |

**Table S3 Comparison of capacity retention of full cells under different current densities with previous works.**

| Additive | Current density (A/g) | Cycle number | Capacity retention (%) | Reference |
| --- | --- | --- | --- | --- |
| DTPA-Na | 1 | 500 | 84.6 | [S12] |
| NH4OAC | 1 | 500 | 83.7 | [S13] |
| D-ZF | 1 | 600 | 82.5 | [S14] |
| GA | 1 | 800 | 65 | [S2] |
| Suc | 1 | 800 | 80 | [S15] |
| GS | 1 | 800 | 89.68 | **This work** |
| ggg | 2 | 900 | 66.5 | [S16] |
| HPA | 2 | 1100 | 80 | [S17] |
| DM-SLS | 2 | 2000 | 81.7 | [S18] |
| GS | 2 | 2000 | 90.42 | **This work** |
| AQS | 5 | 500 | 92 | [S19] |
| FEC | 5 | 700 | 64.4 | [S20] |
| LH | 5 | 1000 | 67 | [S21] |
| C8TAB | 5 | 1000 | 85 | [S22] |
| Ga^3+^ | 5 | 1500 | 78.3 | [S23] |
| ULE | 5 | 1000 | 71.1 | [S24] |
| N-ac | 5 | 2000 | 78.57 | [S25] |
| LA | 5 | 2000 | 80.35 | [S25] |
| EDA | 5 | 2000 | 82.8 | [S11] |
| GS | 5 | 2000 | 95.23 | **This work** |

**References：**

[S1] J. Wan, R. Wang, Z. Liu, L. Zhang, F. Liang, T. Zhou, S. Zhang, L. Zhang, Q. Lu, C. Zhang, Z. Guo, “A Double-Functional Additive Containing Nucleophilic Groups for High-Performance Zn Ion Batteries”, *ACS Nano* 2023, 17, 1610-1621.
[S2] H. Zheng, Y. Huang, J. Xiao, W. Zeng, X. Li, X. Li, M. Wang, Y. Lin, “Multi-Protection of Zinc Anode via Employing a Natural Additive in Aqueous Zinc Ion Batteries”, *Chem. Eng. J.* 2023, 468, 143834.
[S3] H. Wang, W. Ye, B. Yin, K. Wang, M. S. Riaz, B. Xie, Y. Zhong, Y. Hu, “Modulating Cation Migration and Deposition with Xylitol Additive and Oriented Reconstruction of Hydrogen Bonds for Stable Zinc Anodes”, *Angew. Chem. Int. Ed.* 2023, 62, e202218872.
[S4] Z. Jiao, X. Cai, X. Wang, Y. Li, Z. Bie, W. Song, “Trace Amount of Nitrilotriacetate Induced Electrolyte Evolution and Textured Surface for Stable Zn Anode”, *Adv. Energy Mater.* 2023, 13, 2302676.
[S5] W. Deng, Z. Xu, X. Wang, “High-Donor Electrolyte Additive Enabling Stable Aqueous Zinc-Ion Batteries”, *Energy Storage Mater.* 2022, 52, 52-60.
[S6] S. Deng, Y. Sun, Z. Yang, M. Wu, H. Tong, X. Nie, Y. Su, J. Li, G. Chai, “Zwitterion-Separated Ion Pair Dominated Additive-Electrolyte Structure for Ultra-Stable Aqueous Zinc Ion Batteries”, *Adv. Funct. Mater.* 2024, 34, 2408546.
[S7] Q. Zong, R. Li, J. Wang, Q. Zhang, A. Pan, “Tailoring the Whole Deposition Process from Hydrated Zn^2+^ to Zn^0^ for Stable and Reversible Zn Anode”, *Angew. Chem. Int. Ed.* 2024, 63, e202409957.
[S8] Z. Liu, R. Wang, Y. Gao, S. Zhang, J. Wan, J. Mao, L. Zhang, H. Li, J. Hao, G. Li, L. Zhang, C. Zhang, “Low-Cost Multi-Function Electrolyte Additive Enabling Highly Stable Interfacial Chemical Environment for Highly Reversible Aqueous Zinc Ion Batteries”, *Adv. Funct. Mater.* 2023, 33, 2308463.
[S9] L. Liu, X. Wang, Z. Hu, X. Wang, Q. Zheng, C. Han, J. Xu, X. Xu, H. Liu, S. Dou, W. Li, “Electric Double Layer Regulator Design through a Functional Group Assembly Strategy towards Long-Lasting Zinc Metal Batteries”, *Angew. Chem. Int. Ed.* 2024, 63, e202405209.
[S10] Z. Liu, R. Wang, Q. Ma, J. Wan, S. Zhang, L. Zhang, H. Li, Q. Luo, J. Wu, T. Zhou, J. Mao, L. Zhang, C. Zhang, Z. Guo, “A Dual-Functional Organic Electrolyte Additive with Regulating Suitable Overpotential for Building Highly Reversible Aqueous Zinc Ion Batteries”, *Adv. Funct. Mater.* 2024, 34, 2214538.
[S11] L. Deng, X. Xie, W. Song, A. Pan, G. Cao, S. Liang, G. Fang, “Realizing Highly Stable Zinc Anode via an Electrolyte Additive Shield Layer and Electrochemical In-Situ Interface”, *Chem. Eng. J.* 2024, 488, 151104.
[S12] Y. Xia, R. Tong, J. Zhang, M. Xu, G. Shao, H. Wang, Y. Dong, C. Wang, “Polarizable Additive with Intermediate Chelation Strength for Stable Aqueous Zinc‑Ion Batteries”, *Nano-Micro Lett.* 2024, 16, 82.
[S13] D. Han, Z. Wang, H. Lu, H. Li, C. Cui, Z. Zhang, R. Sun, C. Geng, Q. Liang, X. Guo, Y. Mo, X. Zhi, F. Kang, Z. Weng, Q. Yang, “A Self-Regulated Interface Toward Highly Reversible Aqueous Zinc Batteries”, *Adv. Energy Mater.* 2022, 12, 2102982.
[S14] J. Li, Z. Guo, J. Wu, Z. Zheng, Z. Yu, F. She, L. Lai, H. Li, Y. Chen, L. Wei, “Dextran: A Multifunctional and Universal Electrolyte Additive for Aqueous Zn Ion Batteries”, *Adv. Energy Mater.* 2023, 13, 2301743.
[S15] L. Zhou, R. Yang, S. Xu, X. Lei, Y. Zheng, J. Wen, F. Zhang, Y. Tang, “Maximizing Electrostatic Polarity of Non-Sacrificial Electrolyte Additives Enables Stable Zinc-Metal Anodes for Aqueous Batteries, *Angew. Chem. Int. Ed.* 2023, 62, e202307880.
[S16] J. Zhang, Y. Liu, Y. Wang, Z. Zhu, Z. Yang, “Zwitterionic Organic Multifunctional Additive Stabilizes Electrodes for Reversible Aqueous Zn-Ion Batteries”, *Adv. Funct. Mater.* 2024, 34, 2401889.
[S17] Z. Yang, Y. Sun, S. Deng, H. Tong, M. Wu, X. Nie, Y. Su, G. He, Y. Zhang, J. Li, G. Chai, “Amphiphilic Electrolyte Additive as an Ion-Flow Stabilizer Enables Superb Zinc Metal Batteries”, *Energy Environ. Sci.* 2024, 17, 3443-3453.
[S18] H. Chen, H. Meng, T. Zhang, Q. Ran, J. Liu, Ha. Shi, G. Han, T. Wang, Z. Wen, X. Lang, Q. Jiang, “Dynamic Molecular Interphases Regulated by Trace Dual Electrolyte Additives for Ultralong-Life Span and Dendrite-Free Zinc Metal Anode”, *Angew. Chem. Int. Ed.* 2024, 63, e202402327.
[S19] R. Sun, D. Han, C. Cui, Z. Han, X. Guo, B. Zhang, Y. Guo, Y. Liu, Z. Weng, Q. Yang, “A Self-Deoxidizing Electrolyte Additive Enables Highly Stable Aqueous Zinc Batteries”, *Angew. Chem. Int. Ed.* 2023, 62, e202303557.
[S20] D. Xie, Y. Sang, D. Wang, W. Diao, F. Tao, C. Liu, J. Wang, H. Sun, J. Zhang, X. Wu, “ZnF_2_-Riched Inorganic/Organic Hybrid SEI: In Situ-Chemical Construction and Performance-Improving Mechanism for Aqueous Zinc-Ion Batteries”, *Angew. Chem. Int. Ed.* 2023, 62, e202216934.
[S21] Y. Wang, T. Wang, Y. Mao, Z. Li, H. Yu, M. Su, K. Ye, D. Cao, K. Zhu, “Reversible Protonated Electrolyte Additive Enabling Dendrites-Free Zn Metal Anode with High Depth of Discharge”, *Adv. Energy Mater.* 2024, 14, 2400353.
[S22] D. Tang, X. Zhang, D. Han, C. Cui, Z. Han, L. Wang, Z. Li, B. Zhang, Y. Liu, Z. Weng, Q. Yang, “Switching Hydrophobic Interface with Ionic Valves for Reversible Zinc Batteries”, *Adv. Mater.* 2024, 36, 2406071.
[S23] J. Cao, Y. Jin, H. Wu, Y. Yue, D. Zhang, D. Luo, L. Zhang, J. Qin, X. Yang, “Enhancing Zinc Anode Stability with Gallium Ion-Induced Electrostatic Shielding and Oriented Plating”, *Adv. Energy Mater.* 2024, 15, 2403175.
[S24] X. Feng, P. Li, J. Yin, Z. Gan, Y. Gao, M. Li, Y. Cheng, Xi. Xu, Y. Su, S. Ding, “Enabling Highly Reversible Zn Anode by Multifunctional Synergistic Effects of Hybrid Solute Additives”, *ACS Energy Lett.* 2023, 8, 1192-1200.
[S25] D. Xu, X. Ren, H. Li, Y. Zhou, S. Chai, Y. Chen, H. Li, L. Bai, Z. Chang, A. Pan, H. Zhou, “Chelating Additive Regulating Zn-Ion Solvation Chemistry for Highly Efficient Aqueous Zinc-Metal Battery, *Angew. Chem. Int. Ed.* 2024, 63, e202402833.
[S26] R. Jiang, T. Naren, Y. Chen, Z. Chen, C. Zhang, Y. Xie, L. Chen, Y. Qi, Q. Meng, W. Wei, L. Zhou, “Enhanced Hydrogen Bonding Through Strong Water-Locking Additives for Long-Term Cycling of Zinc Ion Batteries”, *Adv. Funct. Mater.* 2024, 34, 2411477.
[S27] J. Li, N. Luo, F. Wan, S. Zhao, Z. Li, W. Li, J. Guo, P. R. Shearing, D. J. L. Brett, C. J. Carmalt, G. Chai, G. He, I. P. Parkin, “Defected Vanadium Bronzes as Superb Cathodes in Aqueous Zinc-Ion Batteries”, *Nanoscale.* 2020, 12, 20638-20648.
[S28] G. Henkelman, H. Jónsson, “Improved Tangent Estimate in the Nudged Elastic Band Method for Finding Minimum Energy Paths and Saddle Points”, *J. Chem. Phys.* 2000, 113, 9978-9985.
